# Hearing temperatures: employing machine learning for elucidating the cross-modal perception of thermal properties through audition

**DOI:** 10.3389/fpsyg.2024.1353490

**Published:** 2024-08-02

**Authors:** Mohr Wenger, Amber Maimon, Or Yizhar, Adi Snir, Yonatan Sasson, Amir Amedi

**Affiliations:** ^1^Baruch Ivcher Institute for Brain Cognition and Technology, Baruch Ivcher School of Psychology, Reichman University, Herzliya, Israel; ^2^Department of Cognitive and Brain Sciences, The Hebrew University of Jerusalem, Jerusalem, Israel; ^3^Computational Psychiatry and Neurotechnology Lab, Department of Brain and Cognitive Sciences, Ben Gurion University, Be’er Sheva, Israel; ^4^Research Group Adaptive Memory and Decision Making, Max Planck Institute for Human Development, Berlin, Germany

**Keywords:** cross-modal correspondences, multisensory integration, sensory, thermal perception, multimodal

## Abstract

People can use their sense of hearing for discerning thermal properties, though they are for the most part unaware that they can do so. While people unequivocally claim that they cannot perceive the temperature of pouring water through the auditory properties of hearing it being poured, our research further strengthens the understanding that they can. This multimodal ability is implicitly acquired in humans, likely through perceptual learning over the lifetime of exposure to the differences in the physical attributes of pouring water. In this study, we explore people’s perception of this intriguing cross modal correspondence, and investigate the psychophysical foundations of this complex ecological mapping by employing machine learning. Our results show that not only can the auditory properties of pouring water be classified by humans in practice, the physical characteristics underlying this phenomenon can also be classified by a pre-trained deep neural network.

## Introduction

The nature of temperature perception and its ties to the physics underlying perceived warmth and coolness has a long and elaborate history. The ancient Greek Democritus proposed a conception of heat and cold based on the presence and amount of spherical fire atoms in the air around us (Berryman, 2004). Following this, the perceived sense of heat was described through the correlation of the phenomena of heat or coolness with the presence or lack of a substance known as phlogiston, and equated with the flow of a fluid substance called caloric (Cajori, 1922). To the present day, the study of thermal properties remains central as more and more comes to be understood about the actual physical properties underlying what we perceive to be heat and cold. And yet even today, regarding precisely *how* we perceive temperature and what it is that is being perceived, the narrative is not nearly so unequivocal.

We gain knowledge about the world through our senses, and there is a timeless delicate balance between how much of our conscious perception is written into our neural makeup like a blank slate from sensory experience with the world and how much is hardwired into our brain from birth (Locke, 1847). The classic view of the senses was one of strict division between them, with information coming from the ears being perceived in the auditory cortex in the brain, information from touch being perceived in the sensory cortex, and so on, with the corresponding routes being largely distinct all the way. It is now becoming clear that this understanding is partial, if not mistaken, as connections continue to be unsurfaced between the senses, for example, between vision and audition (McGurk and MacDonald, 1976; King, 2008; Heimler et al., 2015; MacDonald, 2018), olfaction and gustation (Auvray and Spence, 2008; Spence, 2013, 2015), and so on. The sensations of heat and cold are generally related to somatosensory perception, and consist of a direct tactile interaction with warm or cold substances, that brings about the feeling of hot and cold on our physical body. Temperature perception is considered to be more vague than other sensory experiences. Unlike other senses, the sense of temperature does not have a specific dedicated sensory organ. While sight or hearing can be attributed to distinct organs (eyes, ears), the sense of temperature relies on thermoreceptors that are widely distributed across the skin. Furthermore, the neurological areas underlying thermal sensation are widespread in the brain, and no conclusive primary thermal cortex or area has been recognized (Oi et al., 2017). Thus far, different regions in the insula have been suggested as the best candidates, though the insula is also responsible for various sensations, including pain (Tseng et al., 2010; Peltz et al., 2011; Segerdahl et al., 2015).

Different forms of “thermal senses” are prevalent throughout the animal kingdom, for example snakes are able to “see” and identify their prey by it’s body heat (Campbell et al., 2002). In this study, we took a novel, multifaceted approach to study the multisensory nature of thermal perception in humans and the cross-modal correspondences involved in this form of perception. Several cross-modal correspondences related to temperature have been identified in humans (Spence, 2020). For example, the hue heat hypothesis correlates between the tactile sense and the sense of vision, representing the effect of different colors on subjectively perceived temperature (red-warm, blue-cold) (Mogensen and English, 1926; Ziat et al., 2016). Another correspondence links temperature and auditory pitch (cold-higher pitch) (Wang and Spence, 2017; Spence, 2020).

### Previous related research

We chose to add to the body of work investigating a relatively unexplored (and unexpected) connection - the connection between the physical temperature of pouring water and their recognizable auditory properties in different temperatures (Agrawal and Schachner, 2023). This multisensory connection is particularly intriguing as this correspondence may come about due to perceptual learning through passive daily exposure to liquids of different temperatures. Several research groups have previously conducted studies into various behavioral aspects of this phenomenon. Velasco et al. (2013), asserted that humans can hear the difference between hot and cold liquids pouring, yet they did not attempt to solve the question of how. They also posit that spectral analysis alone cannot account for the difference. A later study showed that the sounds made by pouring water alone are sufficient for individuals to classify the water as hot or cold across different vessel types (Peng and Reiss, 2018). Using the same recordings, another study suggested that this precise ability is dependent upon prior exposure (Agrawal and Schachner, 2023). By comparing the results of children with those of adults, they showed that correlating the sound of pouring water with hot or cold is not innate but acquired by the age of 6 (Agrawal and Schachner, 2023). Yet, there is an ongoing controversy regarding the mechanism underlying this. While this ability is not innate (Agrawal and Schachner, 2023), classic acoustic features have not shown a conclusive characterization of hot versus cold pouring water sounds (Peng and Reiss, 2018). Ultimately, the prior research has concluded that “none of the theory gives an explanation for the temperature dependence in these aspects. In particular, though frequency terms are predicted, their amplitudes are not, yet these amplitudes exhibit the strongest dependence on temperature.” Moreover. they admit that their “work highlights the limitations in theory as well as suggesting directions towards more significant advances (Peng and Reiss, 2018).”

#### Motivation for the study – replication and expansion of previous research

This is where the present study comes in. Through a two part behavioral study, we wished to corroborate first and foremost, the findings indicating that humans are able to differentiate between hot and cold liquids (specifically water) not only through touch but also by audition. Furthermore, we wished to weigh in on the debate regarding whether the ability is innate or acquired and investigate the question of people’s explicit awareness of the different thermal properties represented in the water sounds. Consistent repetition of findings concerning this ability are particularly interesting in themselves as people are highly exposed to these sounds in their daily lives, and yet they are seemingly unaware of how and whether they perceive these properties.

Moreover, and expanding upon previous work, we aimed to establish whether there is a basis for cross modal correspondences (as one has not yet been identified) by testing whether thermal properties can indeed be shown to be physically encoded in the relevant sounds. Finding such physical encoding of the thermal properties would strengthen the understanding that humans are actively perceiving the difference. But what is it about the sounds that allows for such a differentiation? We chose to address the problem from a computational angle under the hypothesis that the successful and consistent classification of pouring water sounds at different temperatures by a machine learning algorithm would indicate a physical mapping of these thermal properties in sound. If there are differences in the physical attributes between different temperatures, yet they do not allow people to consciously perceive the different temperatures of the sounds of water being poured (though they can), it provides an additional cognitive insight. Bringing together machine learning and cognitive science commonly involves either deriving inspiration from cognitive mechanisms to improve computational performance (Adeli and Zelinsky, 2018; Amerineni et al., 2019; Hu et al., 2019) or using DNNs to try and implement suggested models of the human brain (Kell and McDermott, 2019; Drakopoulos et al., 2021; Hausmann et al., 2021). Here, we suggest a third approach in which machine learning is utilized to address the current need for paradigms that build up from observations in the real world. For this purpose, we employed a pre-trained deep neural network (DNN) for characterizing recordings of water at different temperatures being poured. In addition to the machine learning algorithm, to further substantiate and ground the difference in the physical mapping, we employed computational analysis of the auditory features, comparing those of the hot and cold recordings.

## Methods

### Participants

We surveyed 84 participants in the multisensory intuition questionnaire administered online (Age: 33.8 ± 2.79, average ± SD; 54 females, 30 males). A separate group of 53 participants was recruited for the behavioral auditory classification task (Age: 30 ± 2.76; 19 female, 22 male also conducted online). Of these, 12 were excluded due to failure to perform or complete the task (they did not complete all trials, or did not respond in over 10% of the trials). Both studies were approved by the Reichman University IRB committee.

#### Questionnaire

We designed an online questionnaire to assess people’s subjective intuitions concerning their daily experiences with multisensory experiences. Our main purpose was to assess the naive intuition of participants regarding their ability to distinguish the temperature of poured water through sound (“Can you tell if water that is being poured is hot or cold only by hearing it?”). To ensure that participants do not have a contextual and positive bias to the question, we included seven additional dummy questions. Similarly, these questions probed participants’ self-assessment of their abilities to perceive information using an atypical sensory modalities (E.g., “Can you tell if a cup of tea contains sugar or not only by smelling it?,” “Can you tell if a fabric has a smooth texture or a rough texture only by seeing it?”). The questionnaire was presented using Google Forms.

#### Auditory classification task

**Figure 1:**
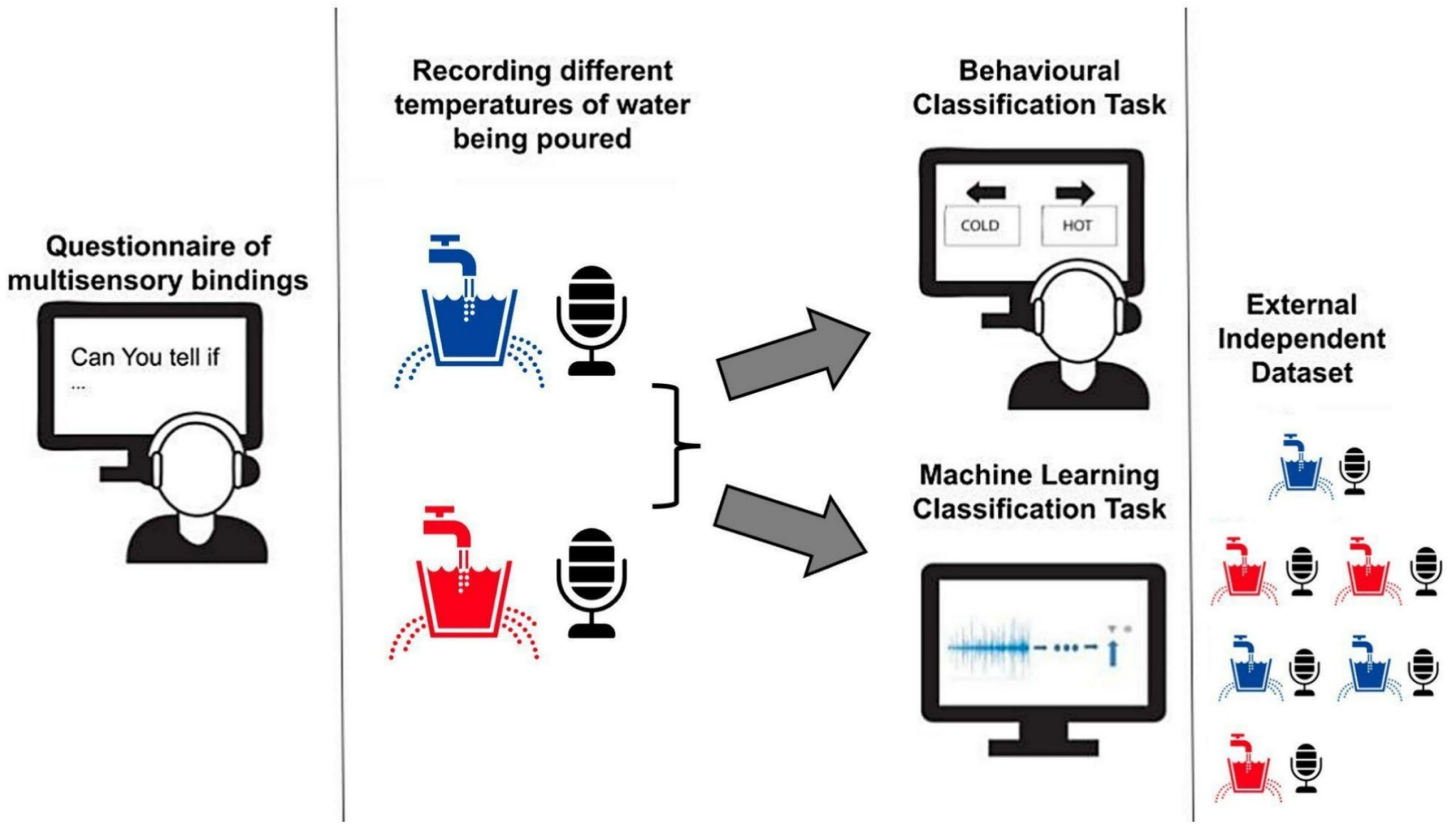
From left to right – we employed a questionnaire to examine whether people think they can tell if pouring water is hot or cold, the vast majority were skeptical. Next, we used recordings of hot and cold water being poured, which were recorded in an acoustic room setting in our lab. We used these recordings to assess the capabilities of people to attribute thermal traits to the sound of pouring water in an online 2AFC task. Finally, we used these same recordings to train a machine learning model to perform the same classification in order to find out if sound physically encodes the thermal traits of pouring water. To avoid potential biases in classification we used an independent test.

In this online experiment, we measured participants’ abilities to classify the temperature of pouring water through sound. First, we instructed participants to wear headphones and adjust their volume to clearly hear an audio sample of water being poured at 35 degrees Celsius (the temperature was disclosed to participants). This sample was used as an auditory calibration and was not part of the experimental stimuli. We implemented a 2-alternative forced choice (2AFC) paradigm in our experimental block. In each trial, participants listened to a single audio recording of poured water, and had to respond if they perceived it to be hot or cold using their keyboard arrows. The block included a total of 25 trials, 20 of which were testing trials while the remaining five were controls aimed to make sure participants were attentive and not merely guessing. Each test trial included one of four possible conditions corresponding to audio recordings of water temperatures at 5^o^C, 10^o^C, 85^o^C and 90^o^ C. Each condition was repeated five times in a randomized order. During the five control trials, no sound was heard, and the participants were instructed to indicate a direction using their right and left keyboard response arrows according to the instructions presented on the screen. The experiment was written in the Unity software (Unity Technologies, version 2020.2.1f1) and presented through the online platform Simmer.io (Simmer Industries) (Figure 1).

#### Stimuli

The acquisition process of the stimuli (water of different temperatures being poured) was highly standardized. The recordings were carried out by one person throughout the acquisition process. The water was poured into one cup throughout, that was kept at room temperature. Prior to pouring, the water temperature was verified to be no more than 0.5 degrees celsius (C) from the desired recorded temperature. A mark was used to ensure that pouring of water commenced from the same initial point for all temperatures, and the pouring rhythm was controlled using a fixed slope and measuring time of 5s to reach the halfway mark of the cup. The recordings were carried out in an acoustically isolated room with a Zoom H6 6-Track Portable Recorder, in stereo with a sampling rate of 44.1 kHz and a bit depth of 24 bits, and were stored in .wav file format.

### Statistical analysis

In analyzing the questionnaire, we measured only the percent of “Yes” responses for the question “Can you tell if water that is being poured is hot or cold only by hearing it?” In analyzing the classification task, all analysis was conducted using Python (pyCharm community edition). Participants’ responses for each sound were categorized by the percentage of “hot” responses (with the percentage of cold responses being complimentary). For within subject analysis, we first compared the cold sounds against each other (5 and 10°C), and the hot sounds against each other (85 and 90°C). We then compared the group of cold temperatures against the group of hot temperatures. While each temperature fits binomial distribution criteria with *p* = 0.5 and there are 10 trials overall, this reaches the criteria to approximate a normal distribution. This requirement is not met for each temperature alone therefore we used the nonparametric Wilcoxon signed rank test. However, This requirement was met by combining both cold and hot temperatures and comparing them against each other (each group includes 10 votes), so this comparison was done using a two-tailed paired t-test. Computational extraction of auditory features was performed using the Librosa library in Python. Mean values for cold and hot temperatures were calculated separately, following which Wilcoxon signed-rank tests were performed to compare the mean features between the cold and hot temperatures.

#### Machine learning procedure

In preparation of the stimuli included in the training set, recordings were acquired similarly to the stimuli in the behavioral experiment, yet pouring of the water continued until the cup was full. A total of 32 audio recordings (16 hot, 16 cold) were obtained with an average length of 9.2 s (average length for cold recordings 9.41 s ± 0.59; for hot recordings 9.06 s ± 0.57). From these 32 files, 15 produced 3 recordings, while 17 produced 2 recordings, resulting in a total of 79 audio segments (40 hot, 39 cold).

The test set consisted of recordings prepared by a research group from Queen Mary University London (QMUL) (Velasco et al., 2013) and included 38 recordings. Nineteen hot recordings of 85°C, and cold recordings of 15°C. Recordings were automated from 5 different fixed heights in range 34–38.7cm and in fixed lengths of 32s each. These were also segmented into four second segments and resulted in 127 labeled audio segments (64 hot; 63 cold).

**Figure 2:**
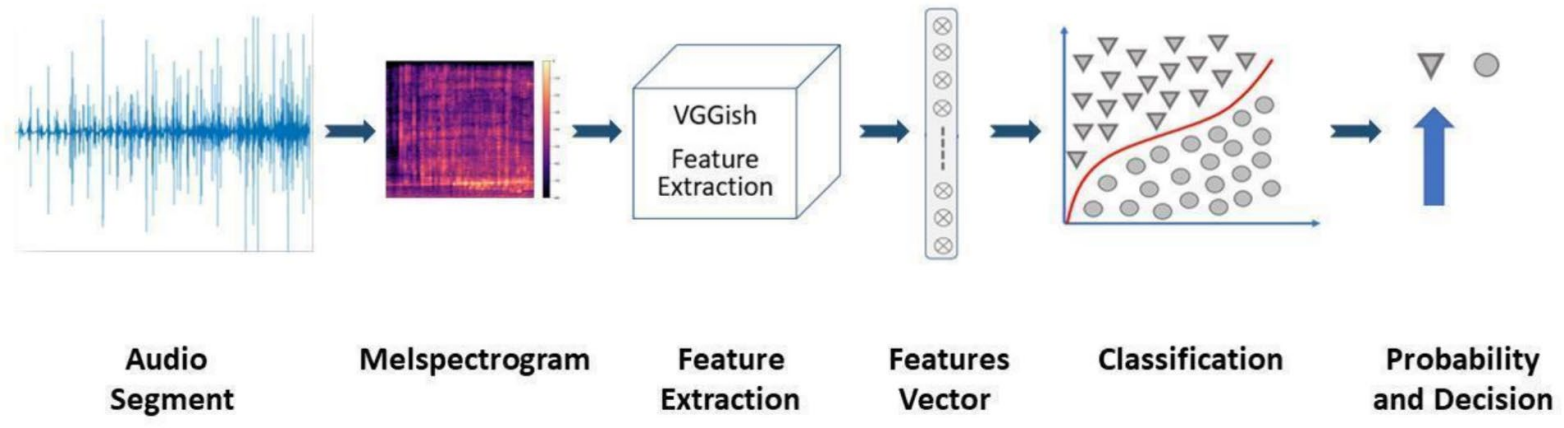
The model flowchart: audio is truncated into segments of 4s; those segments are represented in a Mel spectrogram, so they fit the input for the VGGish net. In the next step the sounds are decomposed using the VGGish feature extractor to a feature vector containing 512 features. These features are fed into the SVM classifier, resulting in a probability score that can be translated into a binary result using a threshold.

The machine learning classifier was trained using a support vector machine (SVM) algorithm, employing a VGGish-based feature extraction module, as described in the VGGish GitHub repository: https://github.com/tensorflow/models/tree/master/research/audioset/vggish. The input to the model is an audio segment, and the output is the probability that the recording is of hot water (cold is supplementary). The probabilities were calibrated using Platt scaling, i.e., running logistic regression on the classifier’s scores, and then fit to a sigmoid whose parameters represent the training data (Figure 2).

The first step involved transforming each audio segment into a Mel Spectrogram (window size of 1,024 samples and hop size of 512 samples, number of Mel bins 64 - covering the range 125–7,500 Hz). The Mel spectrograms were input into the VGGish module for feature extraction, producing 512 features per audio segment. We classified those features using an SVM classifier with a radial kernel (Farhat, 1992).

The model’s hyper-parameters (class weights, regularization, kernel type, and scaling technique), were determined through a 10-fold cross-validation (CV) process (Stone, 1974). Meaning it was trained on 90% of the data and tested on the remaining 10%. This process was run 10 times each time using a different cut of the dataset until all data was part of the test once and the best performing parameters were chosen. A data stratification algorithm was applied to ensure that segments from a single recording would remain in one-fold, thus avoiding data leakage between the train and test sets. Once hyperparameters were chosen, the model was fit by training it on all of the training data.

The results were evaluated using the area under the receiver operating characteristic (ROC) curve. This measures the performance of the model using different thresholds on the output probabilities. The ROC curve indicates the tradeoff between false positives (FP) and true positives (TP). Since the model outputs probabilities, it is possible to determine different thresholds for the binary classification of “hot” (e.g., if a recording is above 0.5 or 0.75 probability of being hot). Hence the area under the curve (AUC) evaluates the overall ability of the model to perform an adequate separation between the classes, in this case hot and cold, regardless of the chosen threshold. In chance level this would be 50%.

For the training set this evaluation was done by averaging the performance of a 10-fold cross validation as described above, and for the testing set this was evaluated using the fit model.

## Results

*Participants claimed that they could not classify the thermal properties of pouring water (hot vs. cold) based on auditory properties*. 80% of participants who responded to the multisensory intuitions questionnaire indicated that they could not differentiate between hot and cold pouring water sounds. The subjects were subsequently asked to explain their response. Among subjects who responded that they cannot tell the difference between hot and cold pouring water sounds, many said that there is no difference in sound, while some stated that there may be a difference in sound, but it is minimal. Among those who responded that they could differentiate, most attributed this ability to a physical difference that leads to variations in auditory properties. Only one participant claimed to have previously noticed the thermal differences represented in sounds. He stated: “I believe I know those sounds, and I remember them differently.”

**Figure 3:**
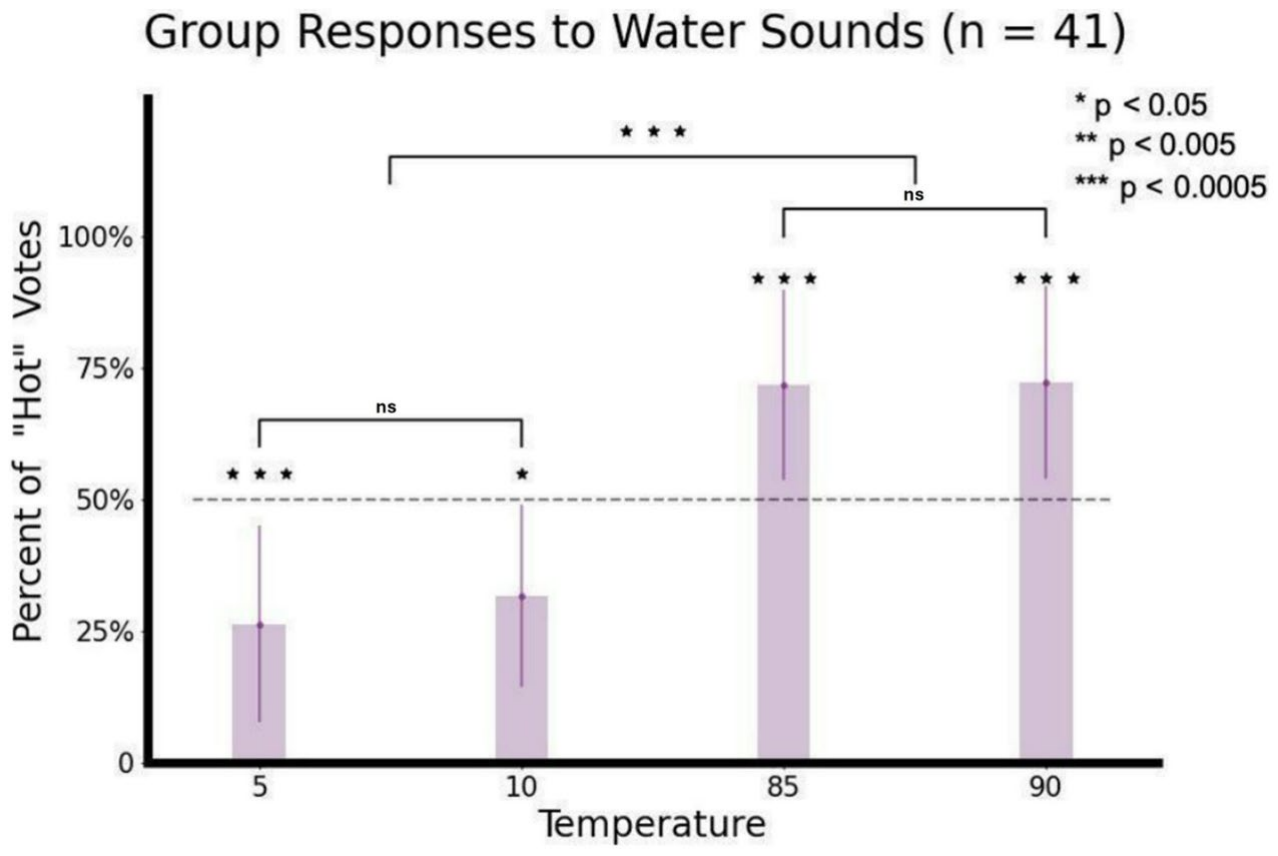
Group “hot” responses in each temperature condition (*n* = 41). Participants were able to correctly classify above chance level each condition temperature by sound alone (two-tailed Wilcoxson signed Rank test). Bunching hot and cold conditions separately and comparing these, we found that the rate of group “hot” responses was significantly higher (two-tailed paired *t*-test). Asterisks denote the significance level of the statistical tests. The black lines indicates the paired comparisons between two conditions or the grouped cold and hot conditions. Error bars show the standard deviation from the mean.

*Participants were able to classify the thermal properties of pouring water (hot vs. cold) based on auditory properties*. Behavioral results are displayed in Figure 3. Results for all temperatures were significantly different from chance level: 5°C (26% + − 18, *r* = −4.37, *p* < 0.0001), 10°C (31% + − 18, *r* = −3.23, *p* < 0.05), 85°C (71% + − 18, *r* = 4.37, *p* < 0.0001), 90°C (72% + − 18 t = 4.75, *p* < 0.0001). Following this, we tested individual differences, by comparing inter-subjects’ responses for rating the hot and cold water sounds. Once we were assured that both cold recordings were perceived as similar (*w* = 0.45, *p* = 0.65) and both hot recordings were perceived as similar (*w* = 0.09, *p* = 0.93), we used a two-tailed paired *t*-test to compare the ratings of each subject. This showed highly significant results (*t* = −6.01, *p* < 0.0001).

**Figure 4:**
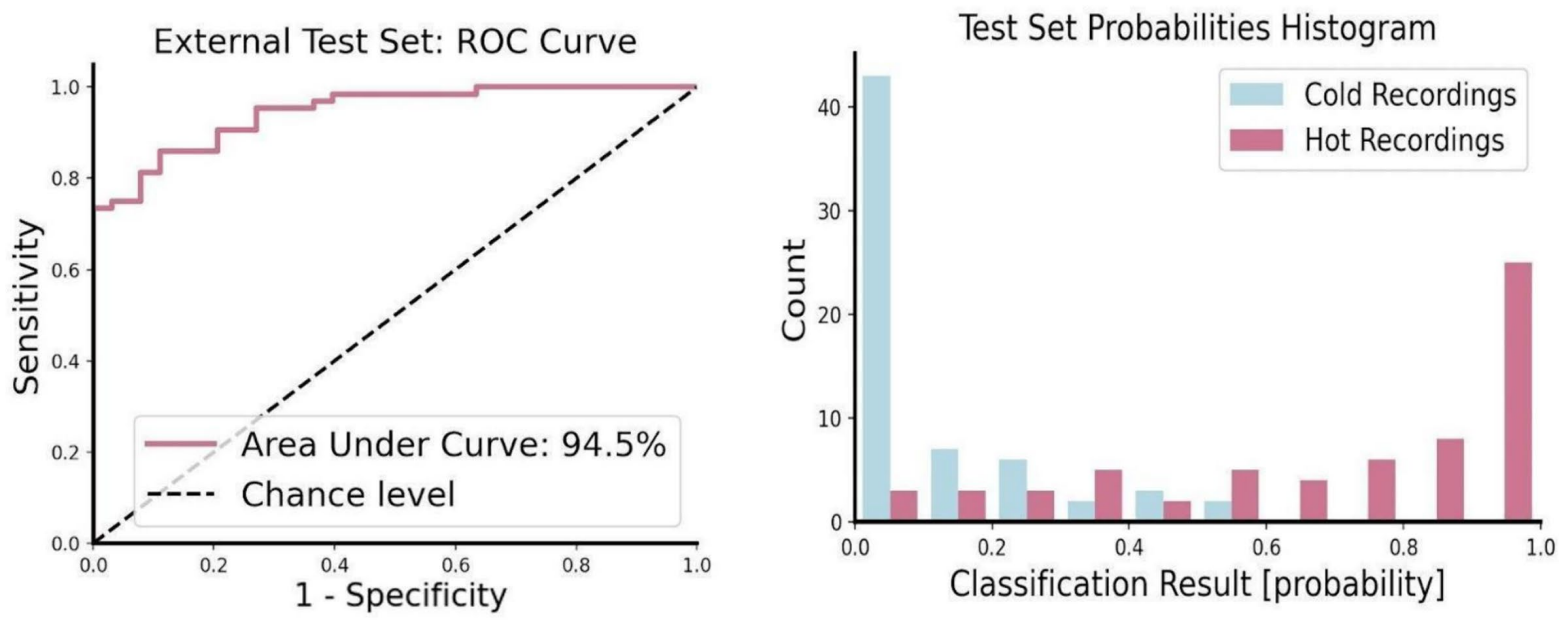
Performance evaluation for the model. **(Left)** This shows the ROC curve that indicates relation between true positive and false positive. The AUC provides an estimation on the model’s overall ability to separate the two given classes. This is a common technique that allows the general evaluation of the model without setting a specific threshold. **(Right)** Histogram of probabilities (the output of the model). The *x*-axis shows the model’s output regarding the probability of a recording to be classified as hot, this ranges from 0 – definitely cold, to 1 – definitely hot. For convenience we drew recordings of hot pouring water in red and recordings of cold pouring water in blue. This figure demonstrates a successful separation between the two classes. We also note that while few hot recordings could be classified as cold the opposite does not occur.

*Thermal traits of the auditory properties of pouring water can be classified by a pre-trained deep neural network*. The DNN was trained on data collected by our group and then tested on data from an external dataset collected by an entirely different group to ensure independence between the training and the testing data. Results on the external test set showed 94.5% AUC, representing a high level of classification ability. (Figure 4) The mean probability of hot recordings was 70% + −30 (*n* = 64). The mean probability for cold recordings was 12% + −14 (*n* = 63), indicating a clear and distinct separation between the two classes. At a later stage, following the validation of the network, we applied the deep learning model to the recordings used for the behavioral experiment to make sure they were not exceptions. Cold recordings’ probabilities were 7% for 5°C and 21% for 10°C, as opposed to hot recordings, with 99.4% for 85°C and 99.7% for 90°C.

*A difference in harmonic properties was revealed by computational extraction of auditory features*. Of the analyses conducted on the extracted auditory features, spectral centroids, zero-crossing rates, and mel frequency cepstral coefficients (MFCCs) did not differ significantly between cold and hot water sounds. On the other hand, chroma features were significantly different between the cold and hot water sounds (*p* < 0.01).

## Discussion

This study aims to shed light on the physical underpinnings of the cross-modal, multisensory nature of thermal perception in humans. The data presented corroborates findings indicating that people are able to perceive the thermal properties of water through audition. More specifically, they are able to discern between hot and cold pouring water by their auditory characteristics. This is despite the fact that the overwhelming majority of people claim that they cannot make precisely this distinction (indicating that the ability is implicitly acquired). By using a different approach - combining a pre-trained DNN with training an SVM algorithm followed by computational extraction of auditory properties, we succeeded in showing that the thermal traits of pouring water are indeed manifested in sound.

As the physical attributes underlying humans’ ability to map auditory properties of thermal traits have remained elusive, we took the novel approach of employing machine learning, more specifically a pretrained VGGish DNN, to shed light on this matter. Our results, namely the ability of the classifier to delineate between the hot and cold pouring water sounds to a high degree of accuracy, point to a physical manifestation of the thermal traits in audition. These findings may strengthen the view that the multisensory link between thermal properties and audition is implicitly learned through passive exposure and perceptual learning. This conclusion, alongside the physical differences we have uncovered between the hot and cold pouring water sounds, has several implications and raises interesting questions.

The last decade has seen an upsurge in the use of machine learning for better understanding observations and phenomena in the real world, which are particularly difficult or elaborate to explore and subsequently generate predictions. This study aims to contribute to this body of knowledge modestly, by showing that there is a physical classification of the thermal properties of pouring water in auditory properties discernable by a machine learning classifier. Previous research had not been successful at achieving these aims. Specifically, the present findings support the view that machine learning could be utilized for providing insight into the current need for paradigms that build up from observation in the real world. In addition, this work takes the approach of using ecological, complex, multidimensional stimuli in the study of perception, an angle that is relatively unexplored in sensory research.

A caveat of this form of study could be that it raises questions at a higher level concerning to what extent machine learning algorithms can constitute underlying computational models with regard to perception and behavior. With regard to perceptual learning, specifically in the visual domain, research findings have shown physiological and behavioral schemas corresponding to those seen in human experiments in trained DNNs (Wenliang and Seitz, 2018; Bakhtiari, 2019). On the other hand, it is well known that in the visual domain, DNNs are “fooled” into classifying images entirely unrecognizable to humans (Nguyen et al., 2015) among other well documented phenomena of DNNs being “fooled” or misclassifying (Szegedy et al., 2014; Zhang et al., 2023). These findings, taken together indicate that while DNNs may represent perceptual learning processes in humans to a certain extent, the question of whether they coincide with the mechanisms taking place in humans when taken to interpret the behavioral level remains to be determined. This is particularly due to the lack of interpretability of current DNNs, which is a limitation of this study. While current DNNs allow us to prove that underlying physical differences exist, as done in the present study, they do not provide the physical correlates themselves. An initial computational extraction of auditory features we conducted uncovered a difference in harmonic properties, specifically chroma features. Further research is thus warranted to provide a more thorough understanding of this perceptual phenomenon.

When employing machine learning to link the physical world to human perception, an important criterion is desired, namely the attainment or corroboration of human performance on the task (Kell and McDermott, 2019). Yet, it is commonly observed that machine learning algorithms differ from the human performance on the task, raising the question of whether and to what extent the underlying performance characteristics match (Jozwik et al., 2017; Kell and McDermott, 2019). This question remains central in machine learning as a whole, as models representing mechanisms underlying vision and audition both improve significantly in performance (Kell and McDermott, 2019), sometimes even outperforming their human counterparts (Geirhos et al., 2021). Insofar as machine learning algorithms of the kind used in the present research are thought to represent underlying performance characteristics faithfully, we feel justified in making the link. In this case, we suggest a correspondence between the findings of the machine learning algorithm and human behavior, possibly strengthening the conclusion that these findings represent a physical manifestation of the thermal traits in audition that are perceived by humans. Yet we cannot discern in this case whether and to what extent the humans outperform the algorithm or vice versa, and such an exploration quantitatively comparing between the two classifications would be warranted in future research.

### Future directions

The results of this study warrant a further extension regarding the question of to what extent humans are capable of differentiating or delineating between the auditory properties of different temperatures. A future study could explore the just noticeable difference (JND) of temperatures perceived through their auditory properties. Previous research into the JND for warm and cold sensations, as represented through the tactile modality on the skin when exposed to environments at different temperatures, suggests that there is a difference between the JND of warm temperatures as compared to cold (Choo, 1998). Following our study, it would be interesting to explore whether these findings are relevant to the perception of temperatures as perceived through audition as well. Our preliminary findings suggest that as represented in audition, differences on the warm end of the spectrum are better perceived than those on the cold end.

Another interesting direction, in light of the implicit perceptual learning supported by this research, would be to provide explicit training on the correspondence between these thermal and auditory properties. From initial research, we have reason to believe that with training, the JND can be further decreased, particularly with relation to warm temperatures as represented by their auditory properties. Following training, it may be possible for humans to perceive slighter differences within the range of temperatures. If this was indeed proven possible, it would indicate that there is in principle auditory information that we are able to use behaviorally but we are not aware of, and so do not actively use. This could also allow for the creation of new sensory experiences, for example, the creation of a sensory transduction system that expands hearing in the human range with the sensing of info or ultrasound. In addition, these fundamental differences in the auditory properties could guide technology connecting infrared sensors to the sounds, enabling sensing of thermal properties without disturbing the visual system - as is the case with thermal vision devices commonly in use at present. While these directions are feasible in theory, there is much to explore before such applications can be applied. The finding that people are able to differentiate between the thermal properties of pouring water is robust, as it has been replicated by numerous studies including our own (Velasco et al., 2013; Agrawal and Schachner, 2023), yet generalization of this phenomenon remains distant at present. Future research should explore not only the nature of the particular auditory properties but also their universality with respect to different materials or properties of liquids, such as viscosity.

Moreover, these findings can further be explored in the brain by conducting neuroimaging studies of people classifying the thermal properties of water by their auditory characteristics, with or without training. Temperature perception in the brain remains relatively poorly defined and understood (Bokiniec et al., 2018), particularly with relation to differentiation in theoretical localization of hot vs. cold sensation (Aizawa et al., 2019). Prior research conducted in our lab has shown that novel topographies can arise in the brain as a result of training (Hofstetter et al., 2021). Research such as this supports the idea that the development and specializations in the brain are task-specific, rather than sensory-dependent (Ricciardi et al., 2014; Cecchetti et al., 2016; Amedi et al., 2017; Sathian and Ramachandran, 2019). Under this interpretation, by employing the principles of perceptual learning, it is possible to induce or enhance the formation of topographies for features not naturally allotted, through nurture, perhaps even those corresponding to novel or enhanced senses (Hofstetter et al., 2021). These future directions and explorations can have significant implications for our understanding of numerous aspects of thermal perception and perception in general, among them the evolution of our perceptual processes, the development of these processes throughout the lifetime, and more.

In conclusion, this study took a mixed approach to studying the nature of temperature perception in humans from a multisensory viewpoint. It serves as a stepping stone to elucidating the connection between the physical properties underlying the temperature of pouring water, and the auditory properties of them that we perceive in our daily interactions with water of different temperatures. First we replicated prior research indicating that this perceptual ability, the “thermal sense,” in humans is acquired without explicit training as people are unaware that they acquire this ability, thereby strengthening the understanding that it is implicitly learned by a process of perceptual learning throughout the lifetime (Velasco et al., 2013; Agrawal and Schachner, 2023). While this prior research, supported by our findings, indicates that humans possess this ability, they have not been able to shed light on why. Expanding on this body of work from a different angle, we showed that these perceptual processes are indeed based on an underlying physical foundation, an encoding of the thermal properties of water in audition that is classifiable by a machine learning algorithm. In addition, we provide a clue as to the nature of these properties through a computational analysis of auditory features that identified a difference in harmonic properties. As opposed to the sterile homogeneous conditions often employed in research on perception, this study adds to the growing field of exploration into multisensory integration with stimuli having high ecological validity, utilizing natural stimuli that are complex and multi-dimensional.

## Data availability statement

The raw data supporting the conclusions of this article will be made available by the authors, without undue reservation.

## Ethics statement

The studies involving humans were approved by the Reichman University School of Psychology IRB. The studies were conducted in accordance with the local legislation and institutional requirements. The participants provided their written informed consent to participate in this study.

## Author contributions

MW: Conceptualization, Investigation, Methodology, Visualization, Writing – original draft, Writing – review & editing. AM: Conceptualization, Project administration, Supervision, Writing – original draft, Writing – review & editing. OY: Conceptualization, Investigation, Methodology, Supervision, Writing – original draft, Writing – review & editing. AS: Conceptualization, Writing – review & editing. YS: Conceptualization, Investigation, Methodology, Writing – original draft. AA: Conceptualization, Funding acquisition, Project administration, Supervision, Writing – original draft, Writing – review & editing.
